# Efficacy and safety of orthokeratology sequentially combined with escalating atropine concentrations for myopia control in children

**DOI:** 10.1038/s41598-025-22722-8

**Published:** 2025-11-06

**Authors:** Ziqi Guo, Zhiyuan Wei, Hongfei Ming, Lijun Zhang, Bing Ma, Yue Zhao, Lei Guo

**Affiliations:** 1 Department of Ophthalmology, Shenyang Sinqi Eye Hospital, Shenyang, China; 2 Ophthalmology and Optometry Centre, The First Hospital of China Medical University, Shenyang, China; 3 The Third People’s Hospital of Dalian, Dalian, Liaoning China; 4 Shenyang Myopia Clinical Medical Research Center, Shenyang, China; 5 School of Optometry, Hong Kong Polytechnic University, Hung Hom, Hong Kong China; 6 Department of Clinical Epidemiology and Evidence-Based Medicine, The First Hospital of China Medical University, Shenyang, China

**Keywords:** Orthokeratology, Low-concentration atropine, Combination therapy, Sequential therapy, Myopia control, Efficacy, Safety, Eye diseases, Refractive errors

## Abstract

To evaluate the concentration-dependent efficacy and safety of sequential orthokeratology (Ortho-K) combined with atropine eye drops (0.01%, 0.025%, 0.05%) for controlling axial elongation in children with progressive myopia, and to characterize clinical outcomes of this stepwise regimen. In this retrospective cohort study, 101 children (199 eyes; age 8–13 years) with axial myopia initially treated with Ortho-K (2020–2024) were analyzed. Participants exhibiting suboptimal responses (axial elongation ≥ 0.15 mm/6 months or ≥ 0.30 mm/12 months) received sequential atropine therapy. Outcomes included axial length changes and adverse event rates across concentration groups. Sequential addition of 0.01%, 0.025%, and 0.05% atropine to Ortho-K reduced annual axial elongation by 28.4%, 31.4%, and 39.4%, respectively (*p* < 0.05 vs. Ortho-K monotherapy). While no severe adverse events occurred, photophobia incidence escalated with higher concentrations (0.05%: 26.3% vs. 0.01%: 8.9%, 0.025%: 11.8%, *p* < 0.001). Near-vision blur occurred exclusively in the 0.05% group (15%). Stepwise atropine escalation following Ortho-K demonstrates clinically meaningful, concentration-dependent myopia control with acceptable tolerability. This sequential strategy provides personalized management for children with rapid progression, balancing efficacy and ocular tolerability.

**Supplementary Information:**

The online version contains supplementary material available at 10.1038/s41598-025-22722-8.

## Introduction

Myopia has emerged as a global public health crisis, with its prevalence escalating rapidly and onset occurring at younger ages^1–3^. Early-onset myopia in children significantly elevates the risk of developing high myopia or pathological myopia later in life, necessitating prolonged management periods and posing greater therapeutic challenges^4–10^. Consequently, myopia control in children and a dolescents has gained urgent societal attention, evolving into a national strategic priority in China^11^.

Currently, there is no cure for pediatric myopia, and clinical efforts focus on curbing its rapid progression^12,13^. Primary interventions include optical, pharmacological, physical, behavioral, and binocular vision training approaches, among which optical and pharmacological strategies are supported by the most robust evidence^14,15^. Optical modalities such as orthokeratology (Ortho-K), defocus-incorporated soft contact lenses,EDOF CL and specialized spectacle lenses (DIMS, DOT lenses, e.g.) are widely utilized^16–18^. Pharmacologically, low-concentration atropine eye drops (0.01%–0.05%) are the most widely studied drug for myopia control, with concentration-dependent efficacy: higher concentrations yield superior myopia control but increased side effects^19,20^. Although the efficacy of 0.01% atropine eye drops remains controversial, this formulation continues to be widely used in clinical practice and is frequently employed in research settings.^21,22^.

Despite these advancements, no single intervetion can completely prevent axial elongation or fully achieve the standard of physiological axial growth when applied alone. Ortho-K alone demonstrates a myopia control rate of 32%–63%^23^, while 0.01% atropine combined with single-vision spectacles shows about 30% efficacy^24^, Some studies even demonstrated no statistically significant difference in the efficacy of prevention and control of axial length compared to placebo^25–27^. This unmet need has driven research into combination therapies. In 2018, Kinoshita et al., pioneered the combination of Ortho-K with 0.01% atropine, reporting significantly enhanced efficacy compared to Ortho-K monotherapy^28^. Subsequent studies have consistently demonstrated that combination therapy significantly improves efficacy compared to monotherapy in certain populations.^29–32^.

However, in clinical practice, a considerable proportion of myopic children still experience rapid axial progression (e.g., > 0.3 mm/year, ATOM)^33^ despite combined treatment with orthokeratology lenses and 0.01% atropine eye drops. To address this issue, we established a sequential therapeutic strategy based on two key studies: one is the concentration-dependent efficacy of low-dose atropine for myopia control demonstrated in the LAMP study^22^. The other is the rapid myopia progression criteria from the ATOM study^33^, following the criteria of ATOM study, we defined axial elongation ≥ 0.15 mm/6 months or ≥ 0.30 mm/12 months as indicating suboptimal control (no-response).The stepwise treatment protocol was implemented as follows: For subjects failing to meet control targets with orthokeratology alone: added 0.01% atropine. For continued progression (≥ 0.15 mm/6 months or ≥ 0.30 mm/12 months) with 0.01% atropine, escalated to 0.025% atropine, maximum concentration: 0.05% atropine (not exceeded).This approach was designed to maximize axial length control efficacy and minimize potential side effects through gradual concentration escalation Provide clinically meaningful response thresholds.To our knowledge, this study presents the first clinical evaluation of concentration-dependent outcomes in sequential Ortho-K-atropine combination therapy.

## Materials and methods

### Patient basic materials and principles of the combined treatment regimen

This study retrospectively reviewed 536 patients (1036 eyes) who underwent fitting of orthokeratology lenses in our hospital from January 2020 to January 2024. All enrolled patients underwent cycloplegic refraction with compound tropicamide before orthokeratology lens fitting. Those with a refractive error of ≤ − 0.50 diopters (D) were diagnosed with true myopia. For patients (non-responders) whose axial length increased by ≥ 0.15 mm within half a year or ≥ 0.3 mm within one year after wearing orthokeratology lenses, they were treated with a combination of 0.01% atropine eye drops (a hospital-made preparation of Shenyang Sinqi Eye Hospital). After the combined treatment, if the axial length still increased by ≥ 0.15 mm within half a year or ≥ 0.3 mm within one year, the treatment was adjusted to a combination of 0.025% atropine eye drops^34^ (purchased by the patient’s parents on their own). After the treatment with the combination of 0.025% atropine eye drops and orthokeratology lenses, if the axial length still increased by ≥ 0.15 mm within half a year or ≥ 0.3 mm within one year, the treatment was adjusted to a combination of 0.05% atropine eye drops (a hospital-made preparation of Changsha Aier Eye Hospital). All three concentration formulations were provided in single-dose, preservative-free vials and were discarded after single use. All axial length measurements were obtained using the IOL-Master-500 ocular biometer (Carl Zeiss Meditec, Germany) following a standardized protocol. Five consecutive measurements were performed for each eye, and the mean value was used for analysis. Both eyes were measured in all participants, with demonstrated measurement stability.

This study was approved by the Ethics Committee of author’s Hospital. The study complied with the principles of the Helsinki Declaration. The parents and legal guardians of the children had fully understood the purpose and methods of this study and provided written consent for the use of data for scientific reasons in this study.

### Enrollment and exclusion criteria

(1) Aged 8–13 years; (2) Refractive error measured with an autorefractor before and after cycloplegia (0. 5% compound tropicamide), and the participants were free of ocular and other systemic diseases affecting visual acuity and myopia progression according to a series of eye examinations; (3) − 1.0D to − 6.00D SER, astigmatism ≧− 2.0DC, anisometropia − 1.00D to − 3.0DS; Both patients with binocular myopia and those with monocular myopia are eligible for enrollment. (4) The 0.01%, 0.025% and 0.05% atropine eye drops are all instilled once a day, one drop each time. Instillation should be carried out at least 15 min before wearing orthokeratology lenses. After instillation, the lacrimal sac area should be pressed for 2–3 min. and no other eye drops should be used within 15 min; (5) Best corrected visual acuity (BCVA, logMAR) ≤ 0 in both eyes; (6) Intraocular pressure (IOP) 10 to 21 mmHg in both eyes; and (7) Follow-up every three months during treatment and complete relevant examinations.

### Ophthalmic examinations

Refraction was performed via an autorefractor (AKR-1, NIDEK, Japan). Best corrected visual acuity was examined with a logMAR visual acuity chart (VSK-VC-Y, Weishikang, Guangzhou, Guangdong, China). Axial length was analyzed with an IOL Master optical biometer (IOLMaster500, Carl Zeiss, Germany). The IOP was examined with a noncontact tonometer (NT-530P, NIDEK, Japan). The amplitude of accommodation (AA) was measured via the push-up method. Corneal staining was visualized via fluorescence staining under a slit lamp with a cobalt blue filter. The corneal status before and after wearing orthokeratology lenses was examined using a Medmont topographer (E300USB, Medmont Internation, Pty Ltd, Australia). The centering, integrity of the defocus ring, and the uniformity of the treatment area were evaluated using the tangential map. Meanwhile, the matching relationship between the pupil and the defocus ring was determined^35^.

### Orthokeratology design

101 cases (199 eyes) met the data requirements and received a combination of Ortho-K and atropine. Among these, 78 individuals (161 eyes) wore VST—designed Ortho-K, and 23 individuals (38 eyes) wore CRT-100/E designed Ortho-K. Optimally designed orthokeratology lenses, such as those with an increased Jesson constant and small—optical—zone—designed lenses, were not included. The positioning of the Ortho-K was centered, and the topography map showed that the defocusing ring was centered or mild to moderate displacement, severe displacement (> 1.0 mm) cannot be included in the group^36^. There were no severe adverse reactions during the wearing period. The matching relationship between the pupil and the defocus ring could be directly observed and recorded on the corneal topographic map. The wearing time of the orthokeratology lenses was 6–10 h every night. During the study period, all lenses were within the valid period (18-month service life), including during concentration adjustments of atropine eye drops. The dynamic and static fitting states of the lenses were observed under cobalt—blue light using a slit—lamp microscope, and the dynamic and static fitting states were good. All enrolled participants underwent the examinations while wearing orthokeratology lenses, with no discontinuation or washout period.

The content of the regarding the questionnaire for the side effects of combination therapy includes: discomfort symptoms after eye—drop instillation, including

Systemic symptoms: headache (yes, no), palpitation (yes, no).

Ocular symptoms:

Photophobia (yes, no) (if yes, whether it is tolerable) (if not tolerable, whether wearing tinted glasses); Difficulty in near—vision (yes, no); Allergic conjunctivitis and blepharodermatitis.

### Statistical analysis

All statistical analyses were performed using SPSS Statistics software (version 22.0; IBM Corporation, Armonk, NY, USA) and GraphPad Prism (version 5.0; GraphPad Software, San Diego, CA, USA). Continuous data are presented as mean ± standard deviation. Comparisons between two groups were performed using independent samples t-tests, while comparisons among multiple groups were conducted using analysis of variance (ANOVA). If statistically significant differences were found, the Least Significant Difference (LSD) method was applied for post-hoc pairwise comparisons. For safety evaluation, paired t-tests were used to compare the differences in intraocular pressure (IOP) and amplitude (AA) at 6 and 12 months from baseline within each group, while inter-group comparisons were still performed using ANOVA. Categorical data are expressed as percentages and were compared between groups using the chi-square (χ2) test.

## Results

### Subject profile

**Fig. 1:**
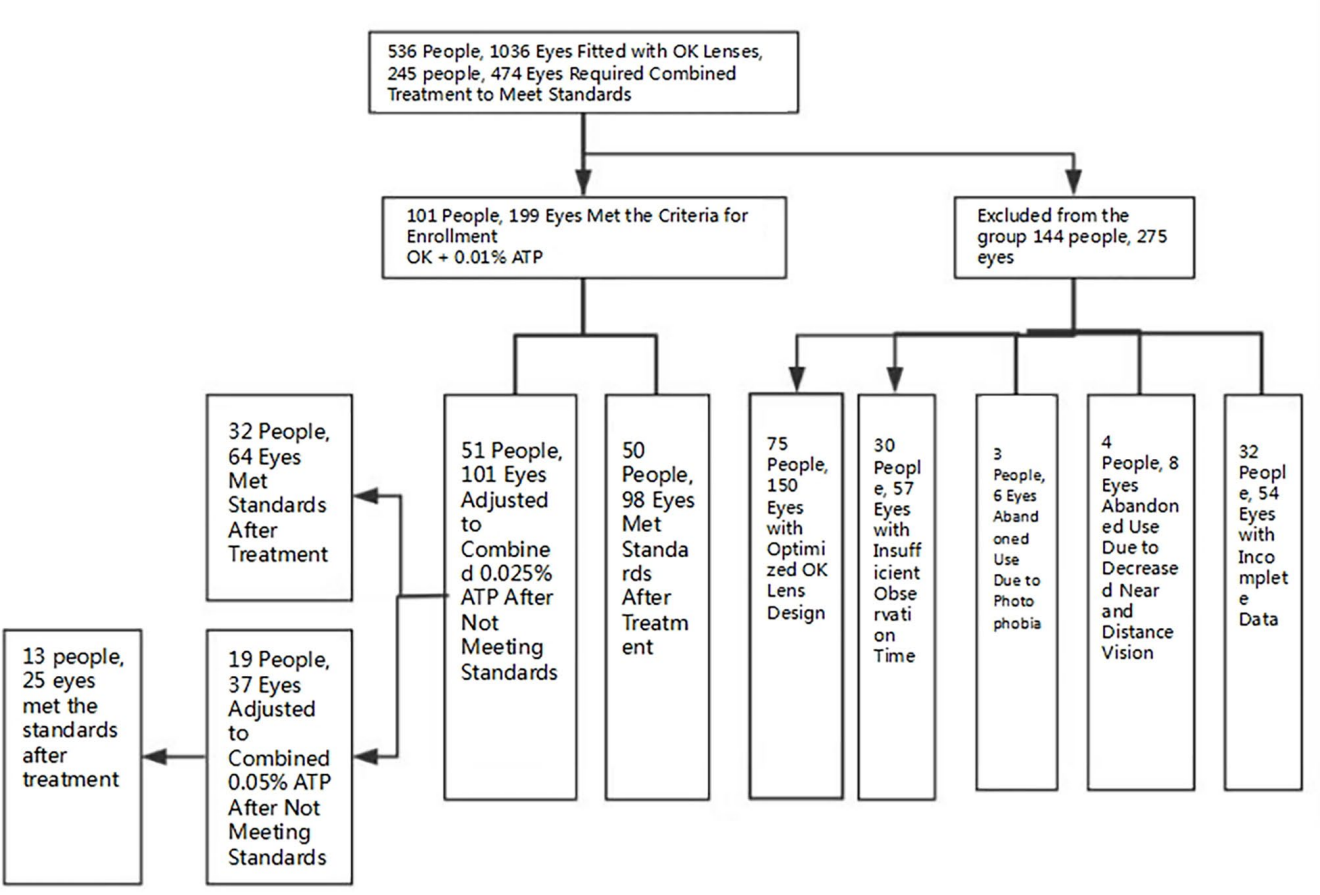
A flow diagram of the study design. OK, orthokeratology lens; OK + 0.01%ATP, orthokeratology lens combined with 0.01% atropine eye drops; OK + 0.025%ATP, orthokeratology lens combined with 0.025% atropine eye drops; OK + 0.05%ATP, orthokeratology lens combined with 0.05% atropine eye drops.

A total of 536 individuals (1036 eyes) were fitted with orthokeratology lenses. Among them, 245 individuals (474 eyes) received combined treatment due to the failure of axial length growth control with orthokeratology lenses alone. 144 individuals (275 eyes) gave up enrollment or combined treatment due to obvious photophobia, blurred vision, Optimize the design of orthokeratology lenses, insufficient observation time as well as incomplete data.as shown in Fig. 1. A total of 101 individuals (199 eyes) were actually enrolled, including 50 males (98 eyes) and 51 females (101 eyes). The average age was 9.0 ± 1.34 years old. The average diopter was − 2.91 ± 0.964 diopters, and the average axial length was 24.5 ± 0.72 mm,please refer to(Table 1).

**Table 1:** Baseline demographic data of all the subjects.

Clinic value	OK	OK + 0.01%ATP	OK + 0.025%ATP	OK + 0.05%ATP	F/χ^2^	*P*
Age (SD)	9.43 ± 1.41	9.43 ± 1.41	8.97 ± 1.09	8.26 ± 0.87	5.37	< 0.001
Man, NO, %	50(50.8%)	50(50.8%)	25(41.2%)	9(47.4%)	1.2	0.753
Female, NO, %	51(49.2%)	51(49.2%)	26(58.8%)	10(52.6%)		
SER (D)	− 2.96 ± 1.15	− 2.96 ± 1.15	− 2.67 ± 0.81	− 2.96 ± 1.15	1.52	0.21
AL (mm)	24.74 ± 0.72	24.74 ± 0.72	24.31 ± 0.71	24.48 ± 0.61	8.2	< 0.001
Genetic history of high myopia	24/101(23.8%)	24/101(23.8%)	18/51(35.3%)	13/19(68.4%)	18	< 0.001

OK, orthokeratology lens; OK + 0.01%ATP, orthokeratology lens combined with 0.01% atropine eye drops; OK + 0.025%ATP, orthokeratology lens combined with 0.025% atropine eye drops; OK + 0.05%ATP, orthokeratology lens combined with 0.05% atropine eye drops. SER, spherical equivalent refraction. AL, axial length. * *P* < 0.05,* *P* < 0.001.

### Changes in axial

**Fig. 2:**
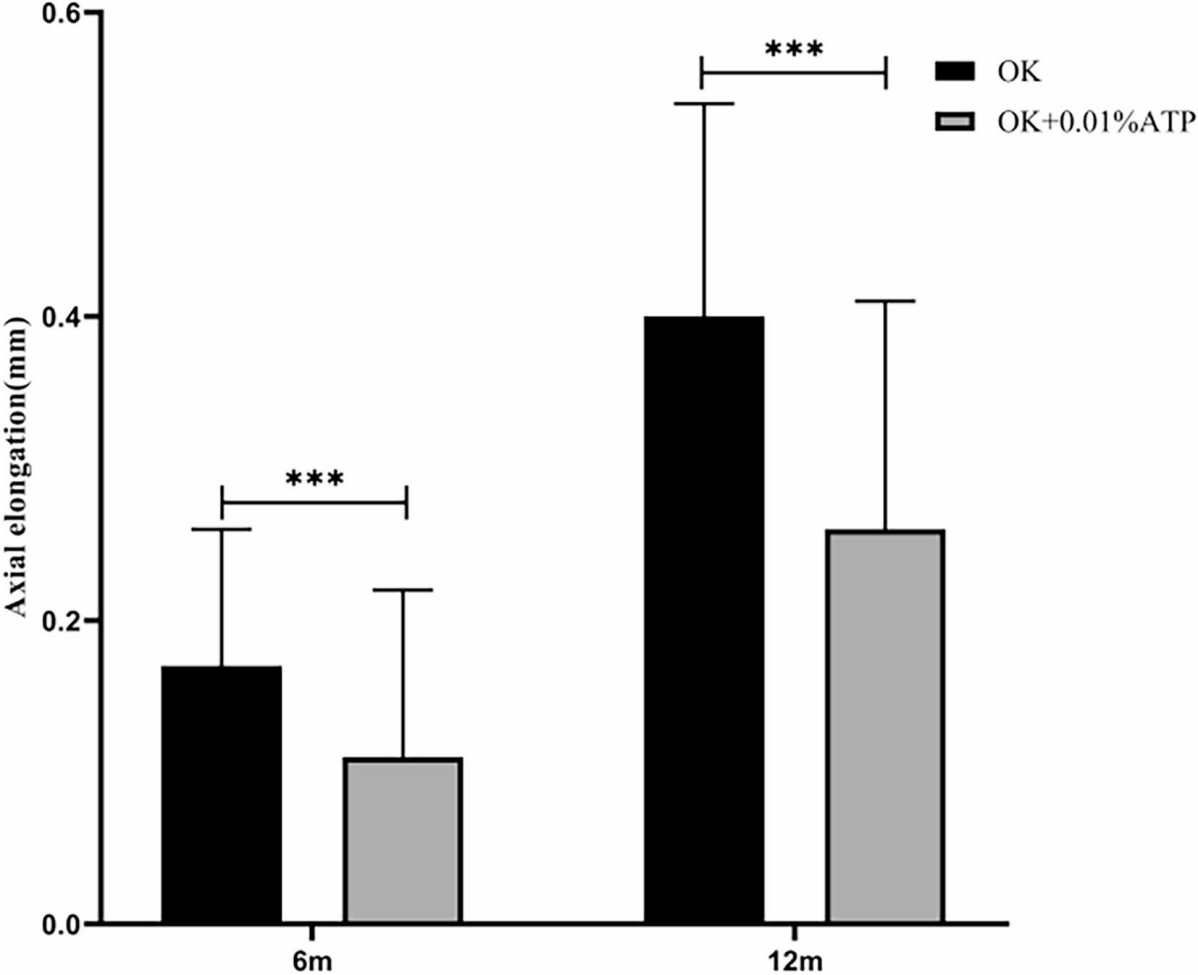
Comparison of axial elongation between orthokeratology lenses combined with 0.01% Atropine Eye Drops and Wearing Orthokeratology Lenses. Axial elongation(mm), OK, orthokeratology lens; OK + 0.01%ATP, orthokeratology lens combined with 0.01% atropine eye drops; * *P* < 0.05, ☨*P* < 0.001.

In subjects showing suboptimal axial length control with orthokeratology (OK) monotherapy, combination therapy with 0.01% atropine eye drops demonstrated significant improvement. The axial elongation at 6 months was 0.17 ± 0.09 mm (OK alone) vs 0.11 ± 0.11 mm (OK + 0.01% ATP) (*p* < 0.001). At 12 months, axial elongation measured 0.40 ± 0.14 mm vs 0.26 ± 0.15 mm respectively (*p* < 0.001), representing a 28.4% reduction in axial growth rate. The proportion of eyes meeting the axial length control target (annual growth < 0.3 mm) was 49.24% (98/199) (Fig. 2, Table 2).

**Table 2:** Comparison of axial elongation between orthokeratology lenses combined with 0.01% Atropine eye drops and wearing orthokeratology lenses alone.

	OK (n = 199)	OK + 0.01%ATP (n = 199)	t	*P*
Axial elongation(mm)/6 m	0.17 ± 0.09	0.11 ± 0.11	5.82	< 0.001
Axial elongation(mm)/12 m	0.40 ± 0.14	0.26 ± 0.15	9.18	< 0.001

OK, orthokeratology lens; OK + 0.01%ATP, orthokeratology lens combined with 0.01% atropine eye drops; Axial elongation(mm).

**Fig. 3:**
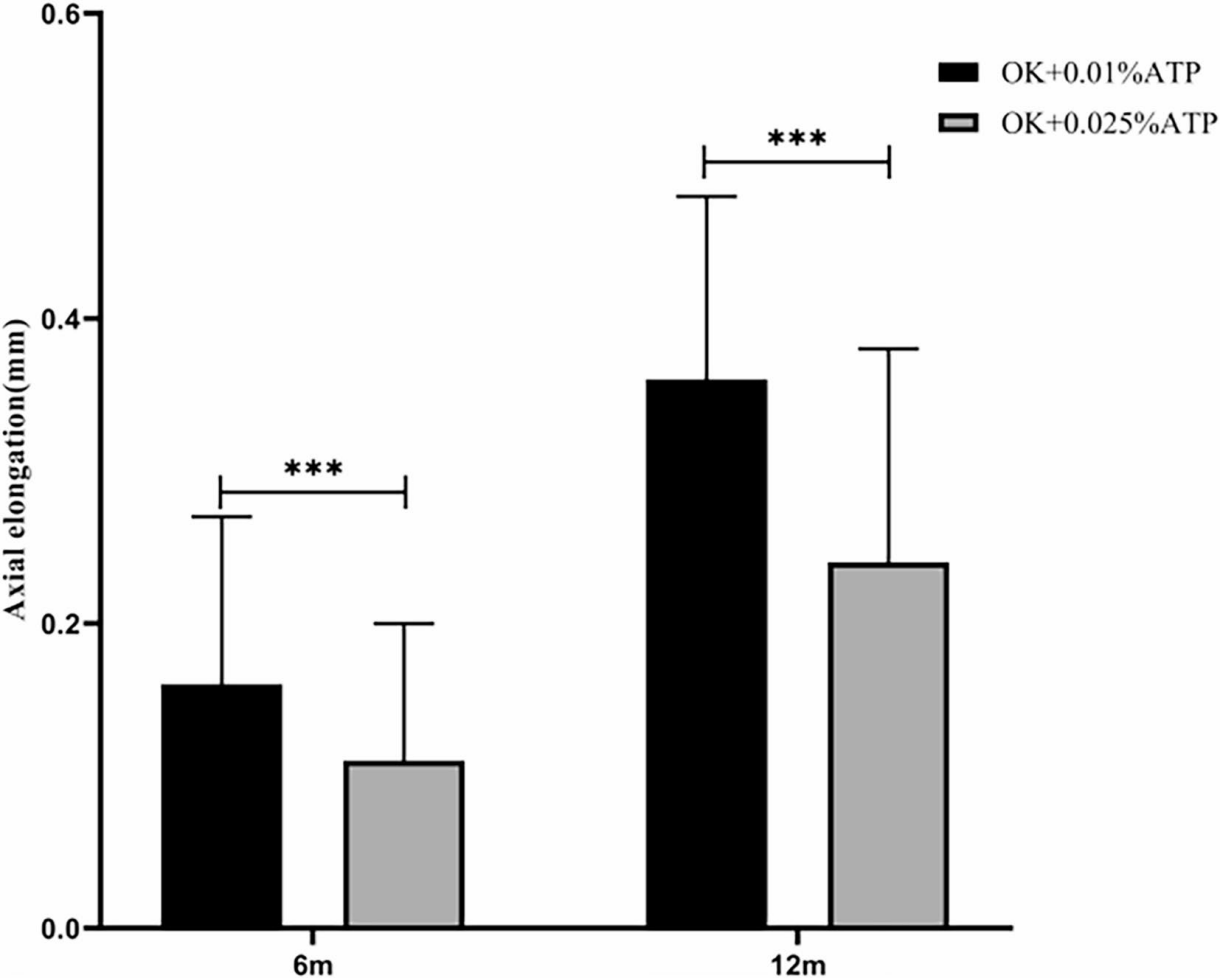
Comparison of axial length growth in patients with suboptimal response to 0.01% Atropine + Orthokeratology Following Addition of 0.025% Atropine.Axial elongation(mm), OK + 0.01%ATP, orthokeratology lens combined with 0.01% atropine eye drops; OK + 0.025%ATP, orthokeratology lens combined with 0.025% atropine eye drops;* *P* < 0.001.

For non-responders to OK + 0.01% ATP therapy, escalation to 0.025% ATP achieved 6-month axial growth of 0.16 ± 0.11 mm (OK + 0.01% ATP)vs 0.11 ± 0.09 mm(OK + 0.025% ATP) (*p* < 0.001), and 12-month growth of 0.36 ± 0.12 mm vs 0.24 ± 0.14 mm (*p* < 0.001), corresponding to a 31.36% reduction. The proportion of eyes meeting the axial length control target (annual growth < 0.3 mm) was 63.36% (64/101) (Fig. 3, Table 3).

**Table 3:** Comparison of axial length growth in patients with suboptimal response to 0.01% Atropine + Orthokeratology Following Addition of 0.025% Atropine.

	OK + 0.01%ATP (n = 101)	OK + 0.025%ATP (n = 101)	t	*P*
Axial elongation(mm)/6 m	0.16 ± 0.11	0.11 ± 0.09	3.55	< 0.001
Axial elongation(mm)/12 m	0.36 ± 0.12	0.24 ± 0.14	6.44	< 0.001

Axial elongation(mm),OK + 0.01%ATP, orthokeratology lens combined with 0.01% atropine eye drops; OK + 0.025%ATP, orthokeratology lens combined with 0.025% atropine eye drops.

**Fig. 4:**
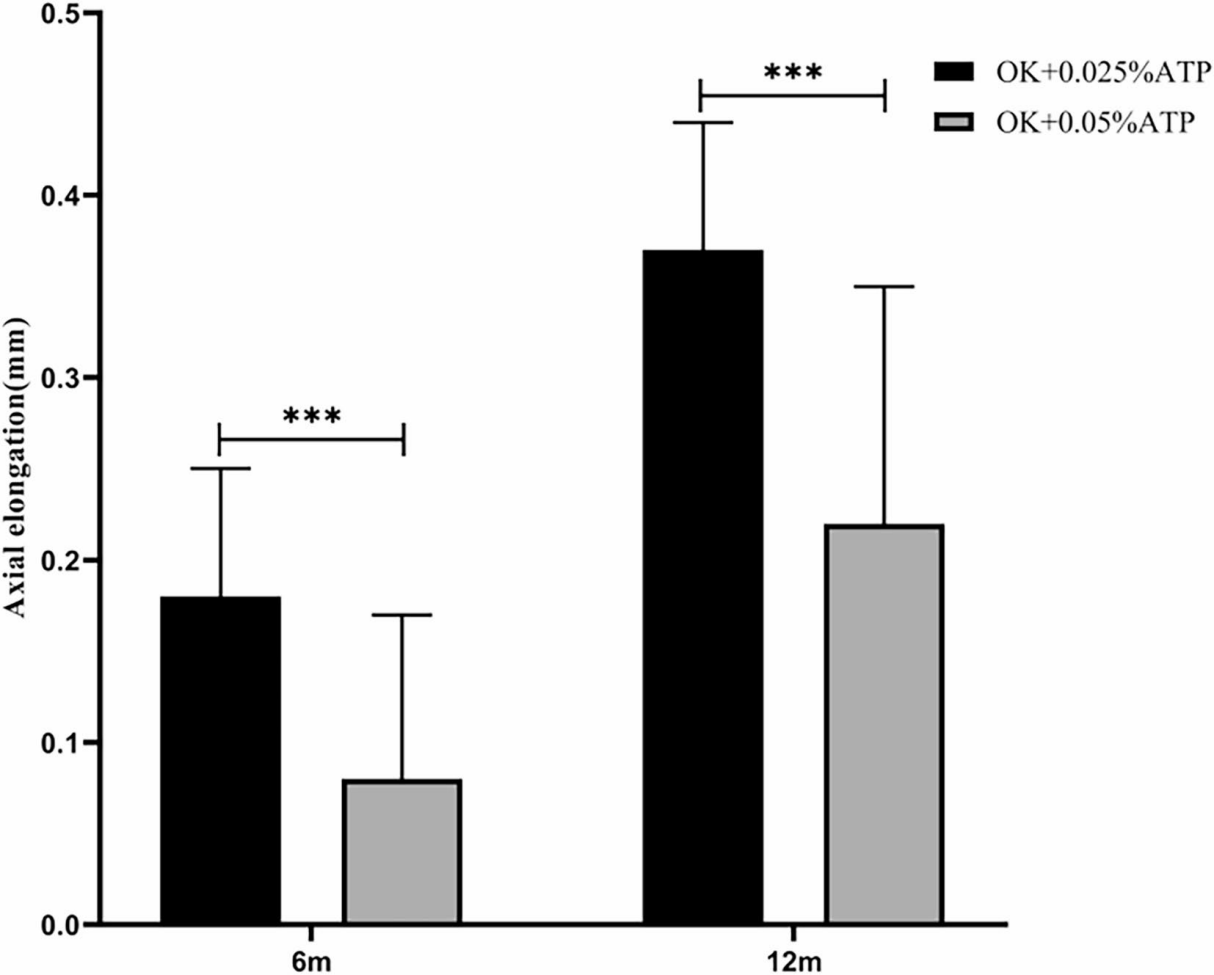
Comparison of axial length growth in patients with suboptimal response to 0.025% Atropine + Orthokeratology. Following Addition of 0.05% Atropine. OK + 0.025%ATP, orthokeratology lens combined with 0.025% atropine eye drops; OK + 0.05%ATP, orthokeratology lens combined with 0.05% atropine eye drops; * *P* < 0.001.

Non-responders to OK + 0.025% ATP showed further improvement with 0.05% ATP: 6-month growth 0.18 ± 0.07 mm (OK + 0.025% ATP) vs 0.08 ± 0.09 mm (OK + 0.05% ATP) (*p* < 0.001), 12-month growth 0.37 ± 0.07 mm vs 0.22 ± 0.13 mm (*p* < 0.001), achieving 39.36% reduction and the proportion of eyes meeting the axial length control target (annual growth < 0.3 mm) was 67.56% (25/37) (Fig. 4, Table 4).

**Table 4:** Comparison of axial length growth in patients with suboptimal response to 0.025% Atropine + Orthokeratology, following Addition of 0.05% Atropine.

	Ok + 0.025%ATP (n = 37)	Ok + 0.05%ATP (n = 37)	t	*P*
Axial elongation(mm)/6 m	0.18 ± 0.07	0.08 ± 0.09	5.36	< 0.001
Axial elongation(mm)/12 m	0.37 ± 0.07	0.22 ± 0.13	6.22	< 0.001

OK + 0.025%ATP, orthokeratology lens combined with 0.025% atropine eye drops; OK + 0.05%ATP, orthokeratology lens combined with 0.05% atropine eye drops; *P* < 0.001.

**Fig. 5:**
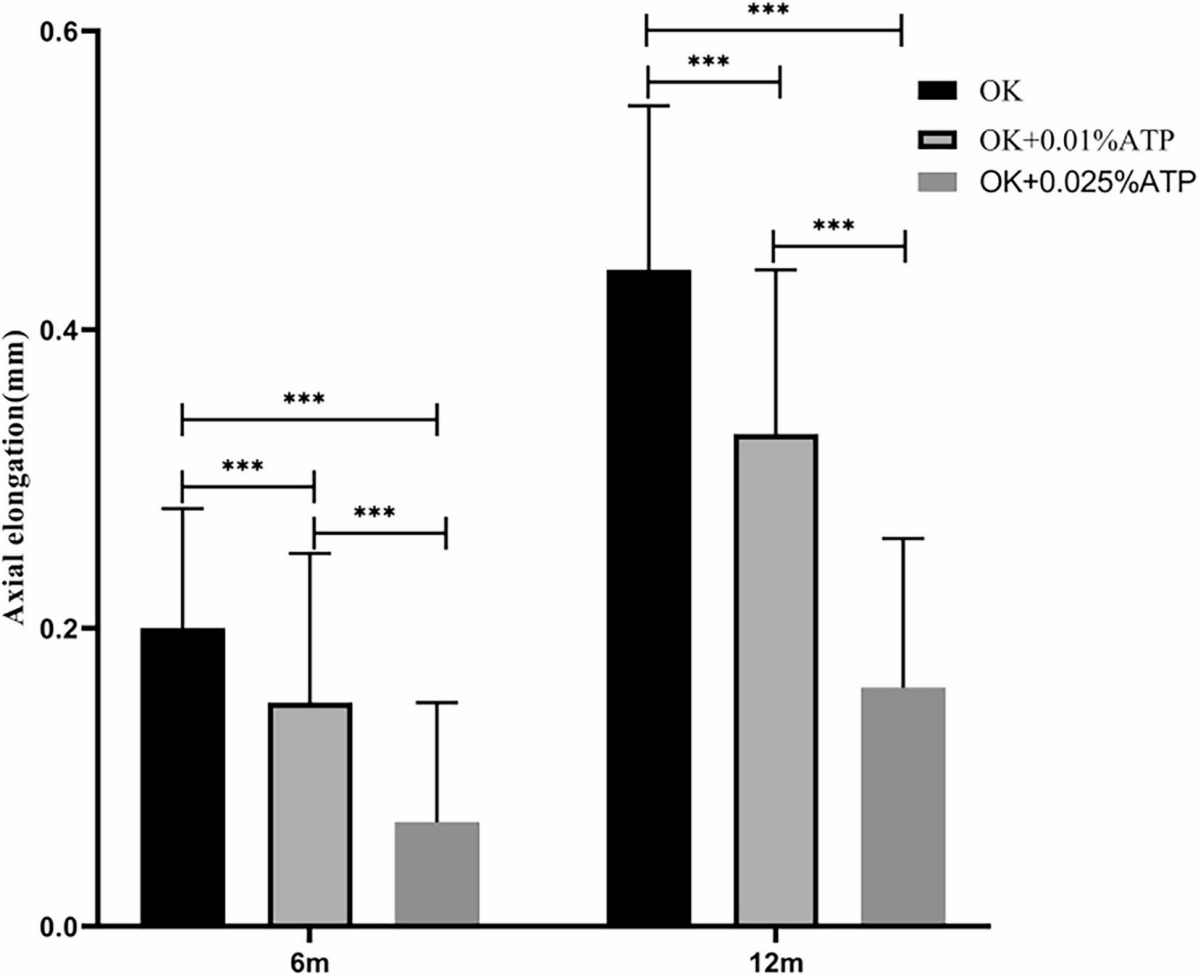
Comparison of axial elongation among patients achieving treatment goals with 0.025% Atropine + Orthokeratology, orthokeratology alone, 0.01%Atropine + Orthokeratology, and 0.025% Atropine + Orthokeratology. The therapeutic effect conforms to the characteristics of concentration dependence.

For individuals who did not achieve the desired therapeutic effect with orthokeratology (OK) lenses combined with 0.01% atropine eye drops, the axial length control efficacy in those who meet the treatment criteria after switching to a combination with 0.025% atropine eye drops. The axial elongation in the groups treated with OK lenses alone, OK lenses combined with 0.01% atropine, and OK lenses combined with 0.025% atropine was as follows: At the 6-month follow-up, the axial elongation was 0.20 ± 0.08 mm, 0.15 ± 0.10 mm, and 0.07 ± 0.08 mm, respectively (OK vs. OK + 0.01% atropine vs. OK + 0.025% atropine). At the 12-month follow-up, the axial elongation was 0.44 ± 0.11 mm, 0.33 ± 0.11 mm, and 0.16 ± 0.10 mm, respectively. The differences among the groups were statistically significant (*p* < 0.001), demonstrating a concentration-dependent therapeutic effect. Detailed results are presented in Fig. 5 and Table 5.

**Table 5:** Comparison of axial elongation among patients achieving treatment goals with 0.025% Atropine + Orthokeratology, Orthokeratology Alone, 0.01%Atropine + Orthokeratology, and 0.025% Atropine + Orthokeratology.

	OK (n = 64)	OK + 0.01%ATP	OK + 0.025%ATP	F	*P*
		(n = 64)	(n = 64)		
Axial elongation(mm)/6 m	0.20 ± 0.08*^#^	0.15 ± 0.10*^&^	0.07 ± 0.08^#&^	36	< 0.001
Axial elongation(mm)/12 m	0.44 ± 0.11*^#^	0.33 ± 0.11*^&^	0.16 ± 0.10^#&^	110.76	< 0.001

Axial elongation(mm),6 m, 6-month; 12 m,12-month; OK, orthokeratology lens; OK + 0.01%ATP, orthokeratology lens combined with 0.01% atropine eye drops; OK + 0.025%ATP, orthokeratology lens combined with 0.025% atropine eye drops, *Representatives of the OK group and OK + 0.01% ATP show a statistically significant difference, # Representatives of the OK group and OK + 0.025% ATP show a statistically significant difference, & Representative OK + 0.01% ATP and OK + 0.025% ATP have statistical differences.

**Fig. 6:**
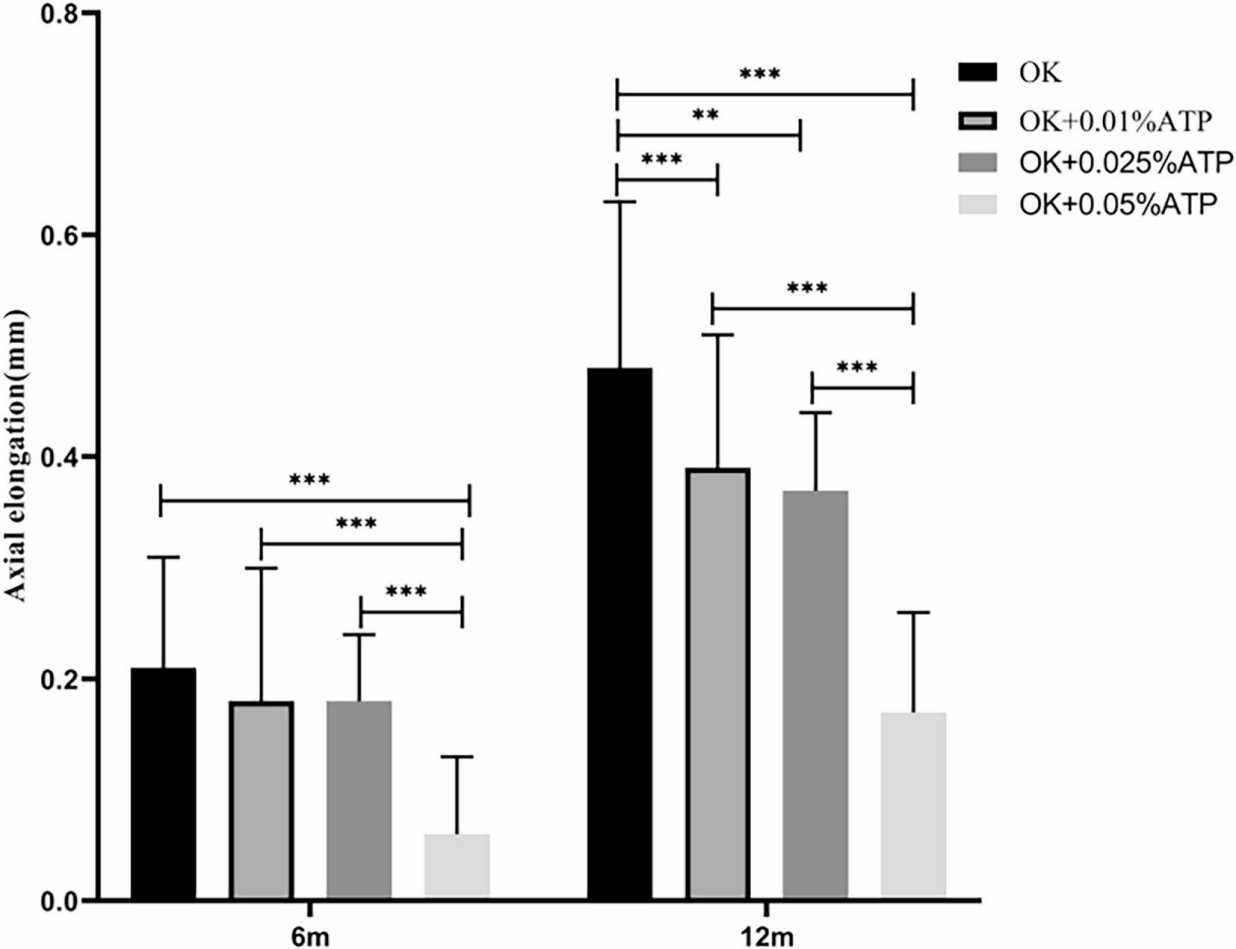
Comparison of Axial Elongation Among Patients Achieving Treatment Goals with 0.05% Atropine + Orthokeratology, Orthokeratology Alone, 0.01%Atropine + Orthokeratology, and 0.025% Atropine + Orthokeratology, 0.05% Atropine + Orthokeratology.

For individuals who did not achieve the desired efficacy with orthokeratology (OK) combined with 0.025% atropine eye drops, the addition of 0.05% atropine eye drops resulted in improved axial length control. The axial elongation in patients treated with OK alone, OK + 0.01% atropine, OK + 0.025% atropine, and OK + 0.05% atropine was as follows: At 6 months, the axial elongation was 0.21 ± 0.10 mm, 0.18 ± 0.12 mm, 0.18 ± 0.06 mm, and 0.06 ± 0.07 mm, respectively (OK vs. OK + 0.01% atropine vs. OK + 0.025% atropine vs. OK + 0.05% atropine). At 12 months, the axial elongation was 0.48 ± 0.15 mm, 0.39 ± 0.12 mm, 0.37 ± 0.07 mm, and 0.17 ± 0.09 mm, respectively.The effectiveness of axial length control was significantly different between the OK + 0.05% atropine group and the baseline, OK + 0.01% atropine, and OK + 0.025% atropine groups (*p* < 0.001). However, there were no significant differences between the baseline, OK + 0.01% atropine, and OK + 0.025% atropine groups (*p* > 0.05). The efficacy did not exhibit a concentration-dependent pattern, as detailed in Fig. 6 and Table 6.

**Table 6:** Comparison of Axial Elongation Among Patients Achieving Treatment Goals with 0.05% Atropine + Orthokeratology.

	OK(n = 25)	OK + 0.01%ATP	OK + 0.025%ATP	OK + 0.05%ATP	F	*P*
		(n = 25)	(n = 25)	(n = 25)		
Axial elongation(mm)/6 m	0.21 ± 0.10*	0.18 ± 0.12^#^	0.18 ± 0.06^&^	0.06 ± 0.07*^#&^	15.5	< 0.001
Axial elongation(mm)/12 m	0.48 ± 0.15*^$@^	0.39 ± 0.12^#$^	0.37 ± 0.07^&@^	0.17 ± 0.09*^#&^	40.08	< 0.001

Axial elongation(mm),6 m,6-month; 12 m,12-month; OK, orthokeratology lens; OK + 0.01%ATP, orthokeratology lens combined with 0.01% atropine eye drops; OK + 0.025%ATP, orthokeratology lens combined with 0.025% atropine eye drops; OK + 0.05%ATP, orthokeratology lens combined with 0.05% atropine eye drops; *P* < 0.001 Representing differences are statistically significant. *Representatives of the OK group and OK + 0.05% ATP show a statistically significant difference, #Representatives of the OK + 0.01%ATP group and OK + 0.05% ATP show a statistically significant difference, & Representative OK + 0.025% ATP and OK + 0.05% ATP have statistical differences, $ representatives of the OK group and OK + 0.01% ATP show a statistically significant difference, @ Representatives of the OK group and OK + 0.025% ATP show a statistically significant difference,

Furthermore, for patients who still did not achieve the desired efficacy with OK combined with 0.05% atropine eye drops, the efficacy did not show a concentration-dependent relationship across the three concentrations, as shown in Table 7.

**Table 7:** Comparison of axial elongation among patients no-achieving treatment goals with 0.05% Atropine + Orthokeratology.

Name/Group	Axial Elongation(mm), 6 m	Axial Elongation(mm), 12 m	Axial Elongation(mm), 6 m	Axial Elongation(mm), 12 m	Axial Elongation(mm), 6 m	Axial Elongation(mm), 12 m	Axial Elongation(mm), 6 m	Axial Elongation(mm), 12 m
	OK	OK	OK + 0.01%ATP	OK + 0.01%ATP	OK + 0.025%ATP	OK + 0.025%ATP	OK + 0.05%ATP	OK + 0.05%ATP
Wang Lu * right	0.24	0.48	0.25	0.38	0.14	0.42	0.28	0.43
Wang Lu *left	0.27	0.56	0.26	0.33	0.31	0.45	0.34	0.47
Lu Zhe * right	0.49	0.66	0.42	0.67	0.24	0.48	0.14	0.33
Lu Zhe * left	0.38	0.52	0.42	0.8	0.26	0.51	0.11	0.25
Yuan Meng * right	0.27	0.56	0.1	0.33	0.21	0.34	0.15	0.46
Yuan Meng * left	0.24	0.47	0.12	0.35	0.2	0.32	0.11	0.37
Wang Ge * right	0.03	0.33	0.14	0.27	0.12	0.41	0.17	0.44
Wang Ge * left	0.13	0.39	0.18	0.4	0.16	0.42	0.21	0.47
Wang Hao * right	0.13	0.34	0.26	0.36	0.25	0.33	0.2	0.3
Wang Hao * left	0.13	0.46	0.21	0.27	0.22	0.31	0.11	0.29
He Jing * right	0.413	0.744	0.42	0.84	0.228	0.456	0.12	0.24
He Jing * left	0.414	0.828	0.36	0.72	0.258	0.516	0.162	0.324

### Safety assesment

In the safety evaluation, no statistically signiffcant differences in IOP were found between the four groups at baseline and follow-up or in the within-group pairwise comparisons between the two follow-up visits and baseline (Table 8). No differences in AA were found among the four groups at baseline, However, after the application of 0.01%, 0.025% and 0.05% atropine eye drops, the AA decreased by − 0.12 ± 0.87D, − 0.45 ± 0.89D and − 1.86 ± 1.30D, respectively, with statistically significant differences compared to the baseline (*p* < 0.05). The reduction in the amplitude of accommodation in the 0.05% atropine eye drops group was significantly greater than that in the other two groups, and the difference was statistically significant (*p* < 0.05) (Table 9).

**Table 8:** Comparison of IOP among patients 0.01%Atropine + Orthokeratology, and 0.025% Atropine + Orthokeratology, 0.05% Atropine + Orthokeratology.

IOP(mmHg)	OK + 0.01%ATP (n = 199)	OK + 0.025%ATP (n = 101)	OK + 0.05%ATP (n = 37)	F	*P*
Basic IOP	16.80 ± 1.74	16.53 ± 1.54	15.53 ± 2.02	5.86	0.004
IOP/6 m	16.59 ± 1.66	16.68 ± 1.54	15.64 ± 1.78	5.14	0.007
IOP/12 m	16.49 ± 1.50	16.64 ± 1.39	15.67 ± 1.88	4.74	0.01
6-month-Baseline	− 0.22 ± 1.44	0.15 ± 1.30	0.11 ± 1.67	0.9	0.41
12-month-Baseline	− 0.32 ± 1.69	0.11 ± 1.33	0.14 ± 1.61	1.22	0.3
P1	0.34	0.35	0.69		
P2	0.24	0.52	0.61

**Table 9:** Comparison of AA among patients 0.01%Atropine + Orthokeratology, and 0.025% Atropine + Orthokeratology, 0.05% Atropine + Orthokeratology.

AA(D)	OK + 0.01%ATP(n = 199)	OK + 0.025%ATP(n = 101)	OK + 0.05%ATP	F	*P*
			(n = 37)		
Basic AA	12.44 ± 1.43	13 ± 1.15	13.47 ± 0.68	13.09	< 0.001
AA/6 m	12.31 ± 1.28	12.59 ± 1.04	11.94 ± 1.13	3.28	0.04
AA/12 m	12.32 ± 1.21	12.55 ± 1.09	11.61 ± 1.10	7.63	0.001
6-month-Baseline	− 0.13 ± 0.78	− 0.41 ± 0.72	− 1.53 ± 1.29	48.48	< 0.001
12-month-Baseline	− 0.12 ± 0.87	− 0.45 ± 0.89	− 1.86 ± 1.30	61.85	< 0.001
P1	0.001	< 0.001	< 0.001		
P2	0.007	< 0.001	< 0.001		

In the questionnaire assessing adverse effects after combined treatment, photophobia was the most common side effect, with incidence rates of 8.91%, 11.76%, and 26.32% for the 0.01%, 0.025%, and 0.05% atropine eye drops groups, respectively. Near vision impairment was only observed in the 0.05% atropine eye drops group, and no cases of allergic conjunctivitis and blepharodermatitis were reported in any of the groups (Table 10).

**Table 10:** Comparison of safety in combined therapy (Questionnaire Results).

Unit: participants	0.01%ATP + OK(n = 101)		0.025%ATP + OK(n = 51)		0.05%ATP + OK(n = 19)	
	n	%	n	%	n	%
Photophobia	9	8.91	6	11.76	5	26.32
Need blackout glasses	0	0	0	0	1	5.26
Difficulty in near—vision	0	0	0	0	3	15.78
Allergic conjunctivitis and blepharodermatitis	0	0	0	0	0	0

No significant systemic or ocular adverse reactions were observed in all enrolled participants. The most common adverse reactions were mild photophobia and blurred near vision, all of which resolved completely after discontinuation of treatment. No statistically significant difference in intraocular pressure was observed before and after the combination therapy (*p* > 0.05). (At 12 months, the difference in IOP between groups was statistically significant but not clinically significant).

### The proportionality relationship between pupil and defocus ring in corneal topograph

**Fig. 7:**
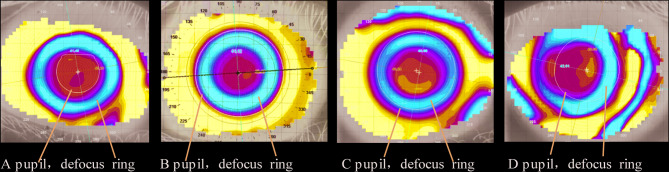
The Proportionality Relationship Between Pupil and Defocus Ring in Corneal Topograph.** A** The pupil is located within the defocus ring.** B** The pupil is located outside the defocus ring.** C** The pupil is tangent to the position of the defocus ring.** D** The defocus ring deviates from the central area.

On the tangential map of corneal topography, the relationship between the defocus ring after orthokeratology lens wear and the pupil can be categorized into four types: the defocus ring located outside the pupil, the defocus ring tangent to the pupil, the defocus ring located inside the pupil, and the defocus ring deviated from the pupil (Fig. 7). With the use of combined 0.01%, 0.025%, and 0.05% atropine eye drops, the proportion of the defocus ring located inside the pupil increased progressively, with rates of 32.4%, 59.1%, 72.5%, and 81.6% for OK, OK + 0.01% ATP, OK + 0.025% ATP, and OK + 0.05% ATP, respectively. The differences were statistically significant (*p* < 0.001). However, there was no statistically significant difference in the deviation of the defocus ring from the pupil (*p* > 0.05) (Table 11).

**Table 11:** The proportionality relationship between pupil and defocus ring in corneal topograph.

Unit:eyes	OK(n = 199)		0.01%ATP + OK(n = 199)		0.025%ATP + OK(n = 101)		0.05%ATP + OK(n = 37)		*P* value
	n	%	n	%	n	%	n	%	
The pupil is located within the defocus ring	76	38.19	45	22.61	8	7.95	0	0	< 0.001
The pupil is tangent to the position of the defocus ring	44	22.11	22	11.06	12	11.88	3	8.1	0.001
The pupil is located outside the defocus ring	64	32.16	117	58.79	73	72.27	31	83.8	< 0.001
The defocus ring deviates from the central area	15	7.54	15	7.54	8	7.9	3	8.1	0.9
Total	199	100	199	100	101	100	37	100	

OK, orthokeratology lens; OK + 0.01%ATP, orthokeratology lens combined with 0.01% atropine eye drops; OK + 0.025%ATP, orthokeratology lens combined with 0.025% atropine eye drops, *P* < 0.001.

## Discussion

Our findings demonstrate that the combination of orthokeratology (OK) lenses with low-concentration atropine eye drops significantly enhances the efficacy of controlling axial length compared to the use of OK lenses alone. The sequential combination of 0.01%, 0.025%, and 0.05% atropine eye drops adheres to the concentration-dependent clinical characteristics of low-concentration atropine, with no serious systemic or ocular adverse effects observed during the treatment. This study indicates that the sequential combination of OK lenses with varying concentrations of atropine eye drops effectively improves the control of myopia progression in children and adolescents, providing a valuable reference for clinical treatment strategies, particularly for cases where monotherapy is insufficient. To our knowledge, this is the first clinical study reporting the sequential combination of OK lenses with different concentrations of atropine eye drops for myopia control in children and adolescents.

Currently, there is no cure for myopia in children and adolescents, and the primary focus of treatment is to control its rapid progression^8,12–17,37^. Despite the increasing number of available control methods, none achieve 100% efficacy, especially when used alone^15–19,37^. Therefore, combination therapy has become a common and effective approach in recent years, particularly for younger children^28–32^. Combination therapy typically involves the use of optical devices alongside pharmacological treatments, with the combination of OK lenses and low-concentration atropine eye drops being a representative example^28–32^. Studies have shown that the efficacy of combination therapy can be additive, with significantly better outcomes compared to monotherapy. Our results indicate that the classic combination of OK lenses with 0.01% atropine eye drops, administered once daily, the rate of axial elongation can be slowed 28.4%. However, this approach still does not completely halt myopia progression, consistent with findings from other studies^22^. While the combination of OK lenses and 0.01% atropine eye drops is more effective than monotherapy, it does not achieve the synergistic effect often rumored in clinical practice (i.e., 1 + 1 > 2).

When the combination of OK lenses and 0.01% atropine eye drops fails to adequately control myopia progression, what should be the next step? Now, there is a lack of literature and evidence-based guidelines to inform this decision. Most meta-analyses on combination therapy focus on the combination of OK lenses and 0.01% atropine eye drops, highlighting a limitation in the current research landscape^28–32^. Wan et al. (2018) and Wen et al. (2025) demonstrated that combining orthokeratology with 0.025–0.125% or 0.05% atropine, respectively, significantly improves axial length control versus monotherapy. However, neither study evaluated 0.01% atropine or conducted dose–response comparisons, leaving the efficacy-safety tradeoffs of lower concentrations unresolved, particularly given the increased side effects associated with 0.05%、0.125% atropine^38,39^. In our study, we addressed this issue by sequentially combining higher concentrations of atropine eye drops, leveraging the concentration-dependent nature of low-concentration atropine. This approach not only improved efficacy but also minimized side effects, offering a new personalized treatment strategy for patients with poor response to lower concentrations.

Although orthokeratology (OK) combined with varying concentrations of atropine eye drops (ATP) generally exhibits a concentration-dependent effect in controlling childhood myopia progression, this rule is not fully followed in clinical practice. In this study, among responders to 0.05% ATP + OK therapy, the expected efficacy gradient between 0.01% and 0.025% concentrations was not observed. Furthermore, in the 6 non-responding patients from the 0.05% ATP + OK group, therapeutic outcomes showed no significant enhancement with increasing concentrations across the 0.01%, 0.025%, and 0.05% ATP treatment arms, demonstrating a departure from typical concentration-dependent characteristics. These patients typically had an early onset of myopia, high initial refractive error, and a family history of high myopia in one or both parents^40–42^.

Low-concentration atropine eye drops exhibit a concentration-dependent effect on myopia control, with higher concentrations offering greater efficacy but also increased side effects. Previous studies have shown that the primary side effects of low-concentration atropine include photophobia and near-vision impairment. The incidence of these side effects is significantly higher with 0.05% atropine compared to 0.025% and 0.01% concentrations. For example, Ji-Sun Moon et al. reported photophobia rates of 3%, 3%, and 14% for 0.01%, 0.025%, and 0.05% atropine, respectively, and near-vision impairment rates of 1%, 2%, and 10%^42^. Our study found higher rates of these side effects, with photophobia occurring in 9.2%, 11.1%, and 25% of patients, and near-vision impairment in 0%, 0%, and 15% of patients, respectively. These findings differ from those of Yam et al. (2019), who reported no significant differences in photophobia and near-vision impairment across the three concentrations and suggested that 0.05% atropine is the optimal concentration^22^. In our study, 7 out of 26 patients in the 0.05% atropine group discontinued treatment due to intolerable photophobia (n = 3) or near-vision impairment (n = 4), whereas no such discontinuations occurred in the 0.01% atropine group. This discrepancy may be attributed to our sequential treatment approach, where patients were exposed to lower concentrations before transitioning to 0.05% atropine, unlike Yam JC’s randomized controlled trial (RCT) design^43^.Moreover, our study employed combination optical therapy, which differs fundamentally from single-modality treatment approaches.

The mechanisms underlying the enhanced efficacy of combination therapy compared to monotherapy remain incompletely understood. Potential mechanisms include: (1) Changes in the relationship between pupil size and the defocus ring. Our results show that increasing the concentration of atropine enlarges the pupil, leading to a higher proportion of pupils that are tangential to or exceed the defocus ring. This change allows the defocus ring of OK lenses to project more effectively onto the peripheral retina, inducing positive peripheral defocus, or “effective defocus”^35,44^. Conversely, when the pupil is too small, the defocus ring may fall outside the pupil, preventing the formation of peripheral defocus or positioning it too far from the macula (e.g., beyond 20 degrees), rendering it ineffective and potentially compromising the myopia control effect of OK lenses^45^. (2) Increased higher-order aberrations with pupil enlargement may also contribute to myopia control, as suggested by studies on defocus spectacles and atropine eye drops^46^. (3) Both OK lenses and low-concentration atropine eye drops can increase choroidal thickness and improve choroidal blood flow, thereby alleviating scleral hypoxia. This effect is further enhanced with combination therapy, representing another potential mechanism for its superior efficacy^47–49^. Although combination therapy may reduce visual quality compared to monotherapy, this reduction could paradoxically contribute to myopia control, as both positive and negative peripheral defocus and astigmatic designs in defocus lenses have been shown to control myopia^50^. As demonstrated by XianLi Du, optimized OK lens designs improve myopia control efficacy at the cost of reduced visual quality, suggesting that sacrificing some visual quality may be a necessary trade-off for effective myopia control^51^.

Our study has several limitations. First, as a retrospective study, the number of fully eligible cases was relatively small due to various confounding factors. Future research should involve larger-scale RCTs. Second, inconsistencies in the sources of atropine eye drops may have influenced treatment outcomes. Third, our study did not include optimized OK lens designs^52–54^, such as those with increased Jessen factors or smaller optical zones, which may further enhance myopia control and reduce the need for higher concentrations of atropine. Fourth, variations in orthokeratology lens designs may introduce some bias to the results. Fifth, the optimal timing for adjusting to the next concentration of atropine eye drops requires further investigation—our cases with 6-month adjustment intervals did not account for potential seasonal influences on myopia progression. Sixth, as myopia progression may slow with increasing age, future studies may benefit from adopting the methodology established by Kaymak, Hakan, et al., which compares the actual annual axial elongation rate against age-matched physiological growth rates, to achieve a more accurate assessment of myopia control efficacy^55^.

## Conclusion

Our study suggests that the sequential combination of OK lenses with varying concentrations of atropine eye drops effectively enhances the control of axial elongation in children and adolescents with myopia, without serious complications. This sequential approach provides new clinical evidence for personalized myopia control strategies.

## Supplementary Information

Below is the link to the electronic supplementary material.

Supplementary Material 1

## Data Availability

All data generated or analysed during this study are included in this published article.
