# Supplementary material for: Efficacy and safety of orthokeratology sequentially combined with escalating atropine concentrations for myopia control in children
Source: Sci Rep. 2025 Nov 6;15:38911. doi: 10.1038/s41598-025-22722-8 (PMC12592541; doi:10.1038/s41598-025-22722-8)
Supplement: Supplementary file 1 — Supplementary Material 1 [file 41598_2025_22722_MOESM1_ESM.docx]

**Adverse Effects Questionnaire for Low-Concentration Atropine Eye Drops**

Name: ___________ Department: ___________ Outpatient ID: __________

**1. Baseline Information**

First-time use: □ Yes □ No

Duration of use: ___________ months

Concentration: □ 0.01% □ 0.025% □ 0.05% □ Other (specify): ___________

Combined therapy: □ Functional spectacles □ Orthokeratology □ Single-vision spectacles

Administration frequency:
□ Once daily
□ Twice daily (morning and evening)
□ Twice daily (evening only)
□ Other (specify): _________

**2.dverse Effects**

Symptoms: □ None □ Present

**2.1 Systemic Symptoms**
□ Dizziness/headache
□ Palpitations/shortness of breath
□ Other (specify): ___________

**2.2 Ocular Symptoms**

Photophobia:
□ Severe (intolerable)
□ Mild (tolerable)
□ Absent

Blurred vision:
□ Near vision
□ Distance vision
□ Both near and distance

Transient irritation:
□ Stinging/burning sensation

Allergic reactions:
□ Eyelid edema
□ Conjunctival chemosis

Dry eye symptoms:
□ Discontinued use due to intolerance
□ Continued use with artificial tears

Other (specify): ___________

**2.3 Symptom Duration**
□ Transient (resolved after instillation)
□ Persistent (duration: ___________ days)

**2.4 Impact on Treatment Continuation**
□ No interruption
□ Treatment discontinued

**2.5 Recurrence upon Re-administration**
□ Not re-administered
□ Recurrence observed
□ No recurrence observed

**2.6 Current Status**
□ Resolved
□ Improved
□ Unresolved

**Parent/Guardian Information**
Name: ___________
Contact number: ___________

**Recorded by**: ___________
**Date**: **/**/___
